# Oral health and self-perceived job readiness among socially disadvantaged and unemployed citizens

**DOI:** 10.2340/aos.v83.42077

**Published:** 2024-10-15

**Authors:** Anna Munk Sigsgaard, Steffen Altmann, Katrine Dannemand Jensen, Børge Hede, Esben Boeskov Øzhayat

**Affiliations:** a Section of Social Medicine, Department of Public Health, University of Copenhagen, Copenhagen, Denmark; b Institute of Labor Economics (IZA) Bonn, Germany; c Department of Economics, University of Copenhagen, Copenhagen, Denmark; d Section of Public Health Dentistry, Department of Odontology, University of Copenhagen, Copenhagen, Denmark

**Keywords:** Unemployment, dental care, vulnerable populations, self-assessment, oral health-related quality of life, return to work

## Abstract

**Objective:**

The aims of this article are (1) to evaluate the association between oral health-related quality of life (OHRQoL) and self-perceived job readiness and (2) to investigate changes in self-perceived job readiness following an oral health promotion intervention.

**Materials and methods:**

The first aim was elucidated in a cross-sectional design, and the second through a prospective intervention study. A survey was administered among 273 unemployed vulnerable people in Copenhagen, Denmark. Participants were randomised to either control or intervention in 2018, and follow-up was conducted 7–15 months later. The intervention entailed support for dental care. OHRQoL was measured by the Oral Health Impact Profile (OHIP-14), and self-perceived job readiness was measured using the Employability Indicator Project (BIP) questionnaire.

**Results:**

The sample reported a high mean OHIP-14 score at baseline (26.9, SD 15.6) and poor OHRQoL was significantly associated with low self-perceived job readiness (*r_s_* = -0.15, *p* = 0.02). The control group reported better job readiness at follow-up compared to the intervention group. However, the effect sizes were small in both groups and no clear pattern was observed.

**Conclusions:**

The results indicate that OHRQoL is linked to self-perceived job readiness. However, the oral health promotion applied in this study did not lead to better self-perceived job readiness. Further research is needed on the effect of oral health promotion interventions on job readiness within socially vulnerable groups.

## Introduction

Poor oral health can have many negative consequences for the individual, including physical and psychosocial impacts [1]. It has further been found that the negative consequences of poor oral health might also affect employment prospects [2]. This has been suggested to be through a mechanism of impaired quality of life, which lowers job-oriented self-efficacy [3, 4]. It is thus surprising that current studies have primarily focused on objectively measured aesthetic aspects of oral health [5, 6] and less on the role of oral health related quality of life (OHRQoL) in relation to employment. This would be relevant as OHRQoL deals with the impact of oral health on quality of life and encompasses both physical and psychosocial aspects beyond aesthetics.

Poor oral health has been found to be especially prevalent in individuals in vulnerable situations, including unemployed people [7–9]. It is thus not surprising that the association between oral health and employment appears to be particularly strong among socially vulnerable people [4, 10, 11]. In this context, it could be hypothesised that unemployed people might improve their employment prospects following dental treatment. A persistent challenge is, however, the inequality in the use of the dental care system, and unemployment has been found to be related to limited use of the dental care system [7, 8].

For this reason, the Danish Government in 2013 introduced a subsidy scheme for dental care through the Act on Active Social Policy (§ 82 and § 82 a) [12]. The Act stipulates that unemployed citizens with no or very limited financial assets can apply the municipality for dental care services performed in private dental practice. The uptake of the subsidy scheme is, however, very low [13], due to a lack of awareness, a highly bureaucratic application process, and psychological barriers towards dental treatment [14]. This calls for new approaches aiming at breaking down these barriers to assist these vulnerable citizens in receiving dental care. In a previous study, we found that an oral health promotion intervention supporting vulnerable unemployed citizens in getting access to dental care increased proximity to the labour market measured objectively using data from a national register on employment [15]. To obtain a broader understanding of the association between poor oral health and unemployment, it is, however, needed to investigate self-reported job readiness.

The aims of this study were therefore (1) to investigate the association between OHRQoL and self-perceived job readiness and (2) to investigate the effect of an oral health promotion intervention on self-perceived job readiness among unemployed vulnerable people. The study will thus add knowledge on the relationship between OHRQoL, dental care, and employment prospects. This is important as this can help to decide if and how oral health programs should be integrated into social and healthcare systems. Our hypotheses are that (1) poor OHRQoL will be associated with low self-perceived job readiness and (2) the oral health promotion intervention will have a positive impact on self-perceived job readiness among the participants.

## Material and methods

### Participants and recruitment

Potential participants were vulnerable unemployed citizens between 18 and 65 years old, affiliated with a job centre in Copenhagen, Denmark. In this study, vulnerable citizens refer to citizens who beyond being unemployed have social issues, such as homelessness, addiction, and crime, and/or health issues, including physical and mental disorders [16]. Further, the citizens have been unemployed for a period of more than 2 years. These citizens are assessed by the municipality to have the potential to return to the labour market after receiving different efforts aiming to overcome their issues. The job centres in Denmark are municipality-run units, with the responsibility for these efforts and guiding unemployed citizens into employment.

Recruitment and randomisation took place at the job centre between April and June in 2018. The research team informed the management in the job centre about the study and intervention, and they communicated this information to the caseworkers who handled the citizens. Further, the caseworkers had written information on the study at their disposal. The citizen was informed about the study and asked about participation when they attended a regular interview at the job centre, which is an interview where the citizen and caseworker discuss the citizen’s progress. If agreeing to participate, the citizen was assigned to either the intervention or the control group by randomisation. The randomisation proceeded according to a schedule made by the research team prior to recruitment. Each calendar day determined the allocation and a similar amount of intervention and control days was ensured. The caseworkers were blinded to accommodate unbiased ascertainment of outcomes. The case workers were selected to handle the recruitment since operating through community partnerships has been found to reduce recruitment challenges among hard-to-reach populations [17]. However, it was not possible to register how many participants were not asked or declined to participate. This was due to difficulties in reaching, motivating, and managing the high number of caseworkers involved, who perceived the recruitment as an extra daily task. This resulted in a lack of registration of participants prior to randomisation. All participants (*N* = 273) received detailed information, signed informed consent, and were then asked to fill out a baseline survey. A follow-up survey was conducted as a telephone interview between January and July 2019 among all the original participants; however, not all participants were reached.

**Figure 1:**
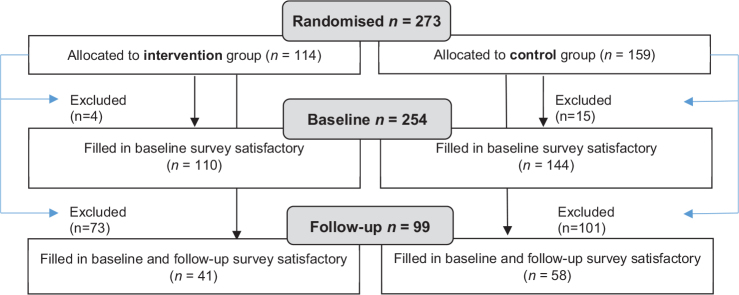
Flow chart of the study sample.

The first aim is elucidated in a cross-sectional design, and the second through a prospective intervention study. To be included in the sample for aim 1 (*baseline sample*), participants had to fill in information on OHRQoL and job readiness in the baseline survey. To be included in the sample for aim 2 (*follow-up sample*), participants also filled in information on job readiness at follow-up. The flow of participants for both aims is depicted in Figure 1. The study was approved by the Danish Data Protection Agency and the Committee on Health Research Ethics (H-17033912).

### Intervention

The goal of the intervention was to improve the possibility of receiving dental care in the target group and thereby improve their job readiness. It thus aimed to overcome two important barriers to receiving dental care for vulnerable people, namely arranging an appointment [18] and having the financial capacity to pay for the care [19]. Overcoming the arrangement barrier was done by a member of the research team giving information on and motivation for dental care. If the participant was in need of and interested in having dental care, the research team offered to make an appointment at a dentist for them on the spot. If this was not done, the participant was instead motivated by the researcher to arrange an appointment. This included information on the benefits of oral treatment for health and quality of life and did not include any type of pressure on the participant. Regarding the financial barrier, the intervention included help from a caseworker to apply for a subsidy for dental care secured in the Danish Act on Active Social Policy (§82 and §82a) [12]. This entailed aiding the participant in retrieving all relevant documents, filling in the application, and sending it to the municipality. The intervention thus did not include any financial support but help and motivation for receiving dental care. The intervention did also not include the actual dental care, which was performed by private practitioners. The intervention was carried out on the day of recruitment. Due to the large amount of documentation required for the application, some participants arranged with the caseworker to return another day to complete the application. The control group was not hindered from applying for subsidy or receiving dental care. If they needed support for applying for a subsidy, they could reach out to their caseworker, who could assist them in the administrative part of the process.

## Methods

Survey data, for the first aim, included information from the baseline survey on OHRQoL and self-perceived job readiness together with information on age, gender, country of birth, cohabitation, and self-rated health status. The same information was used in the second aim together with job readiness from the follow-up survey.

OHRQoL was evaluated using the Oral Health Impact Profile with 14 questions (OHIP-14) administered in the baseline survey [20]. The questions concern problems in the oral region during the past month and were collected in seven domains: functional limitation, pain, discomfort, physical disability, psychological disability, social disability, and handicap. For each question, six response categories and corresponding scores were given: ‘very often’ = 4, ‘fairly often’ = 3, ‘occasionally’ = 2, ‘hardly ever’ = 1, and ‘never’ = 0 constituting a score of 0–56 points, with lower scores indicating better OHRQoL. If answers to five or more items were missing, the questionnaire was discarded. When four or fewer answers were missing, the missing value was imputed using the median score of the participant’s other items. OHIP-14 was kept as a continuous variable in all analyses.

Self-perceived job readiness was measured using the Employability Indicator Project (BIP) questionnaire, which was included in both baseline and follow-up surveys. BIP is a set of indicators, that attempts to measure job readiness among unemployed vulnerable people [16]. Although BIP consists of 11 items, the research team limited the number of questions, by selecting the six items considered to have the greatest relevance in relation to oral health. This was also done to limit the total number of questions in the questionnaire. The six questions concerned how difficult the participant reported it was to (1) initiate contact with strangers, (2) collaborate with others, (3) exert energy to focus on job-related activities, (4) handle health-related issues, (5) handle a job, and (6) know what to do to get a job (Table 2). All items were scored on a five-point Likert scale: 1 = ‘very difficult’, 2 = ‘difficult’, 3 = ‘medium’, 4 = ‘easy’ and 5 = ‘very easy’, which amounts to a total score of 6–30 points with higher scores indicating higher job readiness. If answers to more than three items (>50%) were missing, the questionnaire was discarded. When three or fewer answers were missing, the missing value was imputed using the median score of the participant’s other items.

Information on all covariates was obtained from the baseline survey. All participants were asked in which country they were born, with the following options: ‘*Denmark*’, ‘*Another Western country*’ and ‘*A non-Western country*’. Country of birth was then coded into ‘Denmark (DK)’ and ‘Another country’. Cohabitation status was assessed through the question ‘*What is your cohabitation status?*’: ‘*Living alone*’, ‘*Living with man/woman/girl- or boyfriend*’ or ‘*Other*, *please indicate*’. Response categories were coded into ‘Living alone’ and ‘Living with someone’, including manual recoding of other reported options (e.g. ‘homeless’ and ‘living with children’). Self-rated health status was assessed through the question ‘*In general, would you say your health is?*’ from the Short-Form health survey (SF-12v2) [21]. The response categories were: ‘*Excellent*’, ‘*Very good*’, ‘*Good*’, ‘*Fair*’ and ‘*Poor*’. Response categories were combined into ‘poor/fair’ and ‘Good/Excellent/Very good’.

### Analyses

All analyses were carried out in SPSS statistics version 28, and results were considered significant when the *p*-value <0.05. The normality of continuous variables was assessed through the Shapiro-Wilk test. Descriptive statistics were conducted for all covariates at baseline using Mann–Whitney U and Pearson Chi-Square tests to test for statistical significance of differences between the intervention and the control group. Dropout analyses were performed to investigate differences between included and excluded participants at baseline and follow-up, as the randomisation eventually was lost. For the first aim, the ordinal BIP values were computed into a binary variable for each BIP item (‘very difficult/difficult’ and ‘medium/easy/very easy’). Significant differences in the OHIP-14 mean score across the BIP categories were then evaluated using Mann–Whitney U tests. Spearman’s Rho was performed to assess the correlation between the BIP total score and the OHIP-14 score at baseline. For the second aim, the effect of the intervention on self-perceived job readiness was estimated by calculating BIP pre- and post-intervention scores together with BIP-change scores for each participant by subtracting the baseline score from the follow-up score. The effect was categorised into a binary variable based on the change score (change score >0 = ‘Good effect’ and change score ≤0 = ‘No/poor effect’). The magnitude of the effect was evaluated by the distribution-based methods effect size (ES) [22] and standardised response mean (SRM) [17]. ES was calculated by dividing the mean change score by the standard deviation of the mean baseline score. SRM was calculated by dividing the mean change score by the standard deviation of the mean change score. An effect of 0.2 was considered small, 0.5 moderate and 0.8 large for both estimates.

## Results

Of the 273 participants, 254 (93.0%) were included in the baseline sample, 110 in the intervention group, and 144 in the control group (Figure 1). The follow-up survey was conducted 7–15 months post-baseline to examine aim 2. In total, 99 (36.3%) participants were included in the follow-up sample, 41 in the intervention group, and 58 in the control group (Figure 1).

Table 1 reports baseline characteristics for the baseline and the follow-up samples. In both samples, the majority were women, and the mean age was approximately 47 years. The baseline sample had a lower percentage of people born in DK, fewer cohabitants, better self-rated health, and worse OHRQoL compared to the follow-up sample. Overall, no significant differences in baseline characteristics were seen between the intervention and control groups in the baseline sample. In the follow-up sample, significantly more people were cohabitants in the control group compared to the intervention group (*p* = 0.03). Further, the control group reported better OHRQoL and had higher job readiness at baseline in the follow-up sample compared to the intervention group, though not significant.

**Table 1:** Baseline characteristics of the baseline sample (*n* = 254) and the follow-up sample (*n* = 99) divided into intervention and control group.

	Baseline characteristics																									
	Baseline sample (aim 1)													Follow-up sample (aim 2)												
	All *n* = 254				Intervention *n* = 110				Control *n* = 144				*P*	All *n* = 99				Intervention on *n* = 41				Control *n* = 58				*P*
	*N*	%	Mean	s.d.	N	%	Mean	s.d.	*N*	%	Mean	s.d.		*N*	%	Mean	s.d.	*N*	%	Mean	s.d.	*N*	%	Mean	s.d.	
Women	151	59.4			65	59.1			86	59.7			0.92	59	59.6			25	61.0			34	58.6			0.81
Age			46.9	7.6			47.9	7.5			46.2	7.6	0.05			47.1	7.7			47.0	7.7			47.1	7.7	0.90
Born in DK	137	52.7			56	50.9			81	56.3			0.42	64	64.6			22	53.7			43	72.4			0.06
Living with someone	90	35.4			42	38.2			48	33.3			0.42	63	63.6			21	51.2			42	72.4			0.03
Good self-rated health	72	28.3			29	26.4			43	29.9			0.54	23	23.2			9	22.0			14	24.1			0.80
OHIP-14^a^			26.9	15.6			27.5	15.5			26.5	15.7	0.63			25.2	15.6			24.8	14.0			23.0	16.3	0.56
BIP			16.3	5.4			15.6	5.4			16.7	5.4	0.11			16.4	5.0			15.7	4.5			17.0	5.3	0.20

^a^There were ≤3 missing in the follow-up sample on baseline OHIP-14 (belonging to the control group). OHIP: Oral Health Impact Profile; BIP: Employability Indicator Project.

**Table 2:** Magnitude and distribution of self-perceived job readiness change-effects.

	All				Intervention				Control				*P*
	Mean	s.d.	*n*	%	Mean	s.d.	*n*	%	Mean	s.d.	*n*	%	
BIP total score (*n* = 99)													
Mean baseline score (s.d.)	16.42	5.0			15.66	4.5			16.97	5.3			0.20
Mean follow-up score (s.d.)	16.48	6.1			14.61	5.5			17.81	6.2			0.01*
Mean change (s.d.)	0.06	5.0			-1.05	4.4			0.84	5.3			0.06
ES	0.01				0.23				0.16				-
SRM	0.01				0.24				0.16				-
B1: Initiate contact (*n* = 99)													
Poor/no effect			61	61.6			24	58.5			37	63.8	0.60
Good effect			38	38.4			17	41.5			21	36.2	
B2: Collaborate (*n* = 99)													
Poor/no effect			63	63.6			27	65.9			36	62.1	0.70
Good effect			36	36.4			14	34.1			22	37.9	
B3: Personal energy (*n* = 99)													
Poor/no effect			61	61.6			31	75.6			30	51.7	0.02*
Good effect			38	38.4			10	24.4			28	48.3	
B4: Health (*n* = 99)													
Poor/no effect			75	75.8			34	82.9			41	70.7	0.16
Good effect			24	24.2			7	17.1			17	29.3	
B5: Manage a job (*n* = 99)													
Poor/no effect			70	70.7			26	63.4			44	75.9	0.18
Good effect			29	29.3			15	36.6			14	24.1	
B6: Knowledge of opportunities (*n* = 99)													
Poor/no effect			71	71.7			32	78.0			39	67.2	0.24
Good effect			28	28.3			9	22.0			19	32.8	

* *P* < 0.05. BIP: Employability Indicator Project; SRM: standardized response mean.

In the baseline sample, 19 participants (7.0%) were excluded in the analyses. The results from the first drop-out analysis (results not shown) did not reveal significant differences between the included and the excluded participants across the covariates. In the follow-up sample, 174 participants (63.7%) were excluded, and the drop-out analysis (results not shown) revealed a significantly lower proportion of people born in DK (*p* = 0.003) among the excluded compared to the included participants.

The OHIP-14 mean score was not normally distributed (*p* < 0.001) neither was the BIP total score (*p* = 0.019). The correlation between the OHIP-14 score and the BIP total score at baseline revealed a negative association (*r_s_*= -0.15, *p* = 0.019), indicating a significant but weak association between poor OHRQoL and poor self-perceived job readiness.

**Figure 2:**
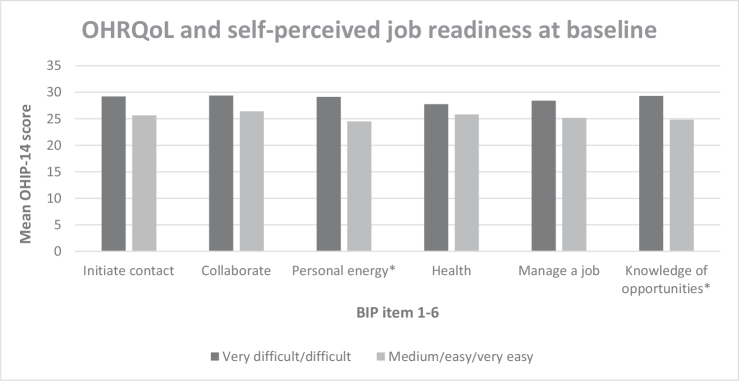
OHRQoL (OHIP-14) and self-perceived job readiness (BIP) measured at baseline. *Significant at *p*-value < 0.05.

For all BIP items, a higher OHIP-14 mean score was seen among participants who reported difficulty with the item compared to participants who reported no difficulty with the item (Figure 2). The difference was significant in item three (*p* = 0.02) and six (*p* = 0.02).

Within the first 6 months after recruitment, 19 (46.3%) in the intervention group and 3 (5.2%) in the control group applied for subsidy. The difference is significant (*p* < 0.001).

The BIP-change score was normally distributed (*p* = 0.11) and the change in self-perceived job readiness following the intervention is illustrated in Table 2 for the whole sample and divided into intervention and control groups. The change in the BIP total score from pre-intervention to post-intervention was higher in the control group compared to the intervention group but not to a significant level (*p* = 0.06). The ES and SRM were small and slightly higher for the intervention group. For the individual BIP items, a significantly higher proportion in the control group experienced a good effect in terms of having the personal energy to focus on getting a job (item 3), compared to the intervention group (*p* = 0.02).

## Discussion

The study sample in general reported poor OHRQoL, which was significantly associated with low self-perceived job readiness, although the correlation was not strong. The oral health promotion intervention applied in this study did not lead to better self-perceived job readiness in the intervention group compared to the control group.

The significant, negative correlation between OHRQoL and self-perceived job readiness was as hypothesised. However, since the association was based on cross-sectional data, we cannot elaborate on the causality of the association. According to the conceptual framework by Singhal et al. [4] exploring the effect of dental treatment on OHRQoL and job-seeking self-efficacy among unemployed vulnerable people, there is reverse causality. Studies should thus examine this in longitudinal study settings to clarify the direction of the association. Another study reported a significant association between OHRQoL and job-seeking self-efficacy [4]. However, the correlation in that study was estimated on changes in scores following dental treatment, which differs from our approach using baseline measures. This might explain the stronger association (*r_s_* = -0.56) found in that study.

Contradictory to our hypothesis, the intervention group did not report better self-perceived job readiness at follow-up compared to the control group. This could be a result of the intervention not having an impact on self-perceived job readiness or the results being affected by differential sample attrition. In the control group, a significantly higher proportion of cohabitants were seen at follow-up compared to the intervention group, which might explain the positive results in favour of the control group, as social relations have been found to be an important determinant of health and well-being [23]. Further, the dropout analyses revealed significantly worse levels of OHRQoL among dropouts, leaving those with the highest treatment need out of the analyses. This could explain the small effect sizes and why the oral health promotion intervention seemingly did not have the expected impact. In the previous study that evaluated the same intervention on objectively measured job readiness, a significant and positive increase was seen in the intervention group [15]. The use of register data for measuring job readiness in that study enabled nearly complete follow-up, which supports our concern about the results in the current study being impacted by differential sample attrition.

Another important aspect to include when discussing our findings is the intervention itself. It targeted the most important barriers in the group in relation to access to dental care. It further took place in the job centre, which is in line with the recommendations in a recent review by Moore and Keat [3]. This means, however, that we did not have the opportunity to secure the citizens actually having dental care, even when scheduling an appointment for them. Therefore, strategies to overcome barriers such as anxiety, lack of transport, and lack of energy were not included in the intervention. Further, even though the intervention aided the citizen in applying for a subsidy, it was not a guarantee that the citizen had completely free dental care. Refinement of the intervention with these aspects could yield a higher effect.

Few other studies have applied oral healthcare interventions in unemployed persons and measured employment-related outcomes. A retrospective cohort study concluded that dental treatment might increase labour market prospects of socially disadvantaged citizens over time, but found no significant differences in employment outcomes between people who underwent dental treatment or not, measured 1 year after treatment [24]. It is thus still debatable whether oral health care interventions and dental treatment lead to better employment prospects for unemployed persons, and more intervention studies are needed to further investigate this [2].

The longitudinal study design and the inclusion of a control group for the second aim are considered two important methodological strengths of this study. Further, the study sample entails socially disadvantaged and unemployed people, who often have limited prospects of returning to the labour market. This emphasises a need for manageable intermediate goals, and we thus also consider the use of self-reported data on job readiness a strength in this study.

Although BIP focuses on indicators that correlate with job probability among socially vulnerable unemployed people, it has some essential limitations [16]. Not all BIP items are able to predict job probability within this vulnerable group, which compromises the internal validity of the study. Another limitation is the relatively long follow-up period we applied, as BIP is designed to be used for more frequent evaluations [16]. Furthermore, not all items were included in this study, which could have resulted in measurement bias on the outcome variable. A relatively high cut-off for missing data was applied before discarding the questionnaire in this study. This was chosen due to the vulnerability of the target group, which had some difficulties in answering all questions. Further, the use of imputation made it possible to include participants with a low number of missing answers. It was thus considered a rationale compromise to obtain relevant knowledge in a group that is difficult to do research in.

Participation in this study was based on a voluntary-driven approach resulting in a skewed distribution of participants in the groups. A high number of dropouts at follow-up affected the study, causing a loss of randomisation in the intervention part of the study, introducing selection bias, and limiting the generalisability. The selection bias was further aggravated by the pre-selection of participants by the caseworkers. This pre-selection could have resulted in not including the most vulnerable people and people not speaking Danish. Unfortunately, it was not possible to register who and how many potential participants were not being asked to participate or declining participation.

Although the included variables are considered relevant, other information with known associations with oral health, such as drug abuse and diet, could have strengthened the study. Further, the lack of information on dental treatment received is also a substantial limitation. This information could have strengthened our process theory, and the comparison to previous findings would have been more straightforward.

Our study does not provide clear evidence on the association between oral health and self-perceived job readiness, and further research is needed on the effect of oral health promotion interventions on self-perceived job readiness among socially disadvantaged people. At the same time, however, it is important that political and governmental decision-makers, who are in the position to make decisions on and enforce legislation in this area, are aware of how oral health is linked to unemployment and that oral health promotion could be justified in terms of reducing health and social inequalities.

## References

[1] Peres MA, Macpherson LMD, Weyant RJ, et al. Oral diseases: a global public health challenge. Lancet. 2019;394(10194):249–260. 10.1016/S0140-6736(19)31146-8. 31327369

[2] Singhal S, Correa R, Quiñonez C. The impact of dental treatment on employment outcomes: a systematic review. Health Policy. 2013;109(1):88–96. 10.1016/j.healthpol.2012.09.016. 23093019

[3] Moore D, Keat R. Does dental appearance impact on employability in adults? A scoping review of quantitative and qualitative evidence. J Br Dent J. 2020 Oct 20; online ahead of print. 10.1038/s41415-020-2025-5. 33082523

[4] Singhal S, Mamdani M, Mitchell A, et al. An exploratory pilot study to assess self-perceived changes among social assistance recipients regarding employment prospects after receiving dental treatment. BMC Oral Health. 2015;15(1):138. 10.1186/s12903-015-0119-2. 26538109 PMC4632367

[5] Halasa‐Rappel YA, Tschampl CA, Foley M, et al. Broken smiles: the impact of untreated dental caries and missing anterior teeth on employment. J Public Health Dent. 2019;79(3):231–237. 10.1111/jphd.12317. 30990228

[6] McErlain M, Newton JT, Jeremiah HG. Does dental appearance affect employment prospects: a prospective cross-sectional study. J Orthod. 2018;45(2):71–78. 10.1080/14653125.2018.1458477. 29637837

[7] Al-Sudani FY, Vehkalahti MM, Suominen AL. Association of current employment status with oral health-related behaviors: findings from the Finnish Health 2000 Survey. Eur J Oral Sci. 2016;124(4):368–376. 10.1111/eos.12276. 27090490

[8] Madero-Cabib I, Reyes C. Employment trajectories across the life course and oral health among older persons in a developing country. J Appl Gerontol. 2022;41(5):1397–1406. 10.1177/07334648211065745. 35050804

[9] Øzhayat EB, Østergaard P, Gotfredsen K. Oral health-related quality of life in socially endangered persons in Copenhagen, Denmark. Acta Odontol Scand. 2016;74(8):620–625. 10.1080/00016357.2016.1229022. 27603026

[10] Bedos C, Levine A, Brodeur JM. How people on social assistance perceive, experience, and improve oral health. J Dent Res. 2009;88(7):653–657. 10.1177/0022034509339300. 19641153

[11] Hall JP, Chapman SLC, Kurth NK. Poor oral health as an obstacle to employment for Medicaid beneficiaries with disabilities. J Public Health Dent. 2013;73(1):79–82. 10.1111/j.1752-7325.2012.00359.x. 22881988

[12] The Danish Ministry of Employment. Bekendtgørelse af lov om aktiv socialpolitik [In Danish] [The active social policy act]. Karnov. Sect. 82. (Accessed on 24th February, 2012). https://www.retsinformation.dk/eli/lta/2012/190.

[13] Skov Kristensen M, Ersbøll AK, Andersen I, et al. Utilization of a public subsidy scheme for dental care services among socially vulnerable citizens out of labor in Copenhagen, Denmark. Acta Odontol Scand. 2023;83:112–119. 10.1080/00016357.2023.2279606. PMC11302627 37938106

[14] Hede B, Thiesen H, Christensen LB. A program review of a community-based oral health care program for socially vulnerable and underserved citizens in Denmark. Acta Odontol Scand. 2019;77(5):364–370. 10.1080/00016357.2019.1572921. 30777469

[15] Sigsgaard AM, Bolvig I, Jensen KD, et al. Oral health promotion and labour market prospects of socially disadvantaged and unemployed people – a randomised controlled trial. Scand J Public Health. 2024;52(1):71–79. 10.1093/eurpub/ckab164.298. 35510343 PMC11328448

[16] Rosholm M, Sørensen KL, Skipper L. What affects job prospects? The employability indicator project. Væksthusets Forskningscenter: Væksthusets Forskningscenter; 2019. p. 41. (Accessed on 01st July 2021). http://vaeksthusets-forskningscenter.dk/wp-content/uploads/2019/11/What-affects-job-prospects.pdf.

[17] Bonevski B, Randell M, Paul C, et al. Reaching the hard-to-reach: a systematic review of strategies for improving health and medical research with socially disadvantaged groups. BMC Med Res Methodol. 2014;14(1):42. 10.1186/1471-2288-14-42. 24669751 PMC3974746

[18] Stormon N, Sowa PM, Anderson J, et al. Facilitating access to dental care for people experiencing homelessness. JDR Clin Trans Res. 2021;6(4):420–429. 10.1177/2380084420952350. 32853528

[19] Palència L, Espelt A, Cornejo-Ovalle M, et al. Socioeconomic inequalities in the use of dental care services in Europe: what is the role of public coverage? Community Dent Oral Epidemiol. 2014;42(2):97–105. 10.1111/cdoe.12056. 23786417 PMC3864569

[20] Slade GD. Derivation and validation of a short-form oral health impact profile. Community Dent Oral Epidemiol. 1997;25(4):284–290. 10.1111/j.1600-0528.1997.tb00941.x. 9332805

[21] Ware JE, Kosinski M, Keller SD. SF-12: how to score the SF-12 physical and mental health summary scales. Boston: The Health Institute, New England Medical Center; 1995.

[22] Cohen J. Statistical power analysis for the behavioral sciences. 2nd ed. Hillsdale, NJ: L. Erlbaum Associates; 1988. 24 p.

[23] Tanggaard Andersen P, Holst Algren M, Fromsejer Heiberg R, et al. Social network resources and self-rated health in a deprived Danish neighborhood. Health Promot Int. 2018;33(6):999–1009. 10.1093/heapro/dax051. 28973140

[24] Singhal S, Mamdani M, Mitchell A, et al. Dental treatment and employment outcomes among social assistance recipients in Ontario, Canada. Health Policy. 2016;120(10):1202–1208. 10.1016/j.healthpol.2016.08.011. 27639285

